# Hamartomatous polyposis syndromes

**DOI:** 10.1186/1897-4287-11-4

**Published:** 2013-06-01

**Authors:** Zoran Stojcev, Pawel Borun, Jacek Hermann, Piotr Krokowicz, Wojciech Cichy, Lukasz Kubaszewski, Tomasz Banasiewicz, Andrzej Plawski

**Affiliations:** 1 Department of General, Vascular and Oncologic Surgery, Regional Specialistic Hospital, Slupsk, Poland; 2 Department of Oncologic Surgery, Gdansk Medical University, Gdansk, Poland; 3 Institute of Human Genetics, Polish Academy of Sciences, Strzeszynska 32, Poznan, 60-479, Poland; 4 Department of General Surgery, Gastroenterological Surgical Oncology and Plastic Surgery, Poznan University of Medical Sciences, Poznan, Poland; 5 Department of General and Colorectal Surgery, Poznan University of Medical Sciences, Poznan, Poland; 6 Department of Pediatric Gastroenterology and Metabolic Diseases, First Chair of Pediatrics, University of Medical Sciences, Poznan, Poland; 7 Fourth Clinical Hospital, University of Medical Sciences, Poznan, Poland

**Keywords:** Juvenile polyposis syndrome, Peutz-Jeghers’ syndrome, Hereditary mixed polyposis syndrome, Cowden’s syndrome, BMPR1A gene, SMAD4 gene, PTEN gene, STK11 gene

## Abstract

Hamartomas are tumour-like malformations, consisting of disorganized normal tissues, typical of the site of tumour manifestation. Familial manifestation of hamartomatous polyps can be noted in juvenile polyposis syndrome (JPS), Peutz-Jeghers’ syndrome (PJS), hereditary mixed polyposis syndrome (HMPS) and *PTEN* hamartoma tumour syndrome (PHTS). All the aforementioned syndromes are inherited in an autosomal dominant manner and form a rather heterogenous group both in respect to the number and localization of polyps and the risk of cancer development in the alimentary tract and other organs. Individual syndromes of hamartomatous polyposis frequently manifest similar symptoms, particularly during the early stage of the diseases when in several cases their clinical pictures do not allow for differential diagnosis. The correct diagnosis of the disease using molecular methods allows treatment to be implemented earlier and therefore more effectively since it is followed by a strict monitoring of organs that manifest a predisposition for neoplastic transformation.

## Introduction

The term of hamartoma corresponds to a non-neoplastic tumour, consisting of disorganized normal tissues, typical of the site of tumour manifestation. The term was introduced by the German pathologist, Eugen Albrecht in 1904 [1]. Familial manifestation of hamartomatous polyps can be noted in a number of morbid syndromes. The diseases include juvenile polyposis syndrome (JPS), Peutz-Jeghers’ syndrome (PJS), hereditary mixed polyposis syndrome (HMPS) and *PTEN* hamartoma tumour syndrome (PHTS). It has also been suggested that the complex includes, i.a., Cowden’s syndrome (CS), Bannayan-Riley-Ruvalcaba syndrome (BRRS), Proteus’es syndrome (PS). All the aforementioned syndromes are inherited in the autosomal dominant manner and are conditioned by mutations in four genes (Table 1). Besides the manifestation of hamartomatous polyps in the alimentary tract these infrequent syndromes are characterized by an increased risk of neoplastic transformation. Development of neoplastic lesions is not restricted to the gastrointestinal tract, it may also involve other organs. The progression of neoplastic lesions in polyps of the type has not been fully recognized but the involved mechanism of neoplastic transformation is distinct from that documented in adenomas (Table 2).

**Table 1:** Genes preconditioning syndromes of hamartomatous polyposis

**Gene**	***BMPR1A***	***SMAD4***	***PTEN***	***STK11***
Name	Bone morphogenetic protein receptor, type Ia	Mothers against decapentaplegic, drosophila, homolog of, 4	Phosphatase and tensin homologue	Serine/threonine protein kinase 11
Function of the protein	receptor	signalling protein	phosphatase	kinase
MIM	*601299	*600993	*601728	*602216
Chromosome	10q22.3	18q21	10q23.31	19q13.3
Size	168.5 kbp	38.5 kbp	108 kbp	23 kbp
Exons	13	11	9	10
Amino acids	532	552	403	433
Transcript (bp)	3613	8365	*9007*	3276
Mass of protein	60 kDa	60 kDa	47 *kDa*	48.6 kDa

An asterisk (*) before MIM entry number indicates a gene.

**Table 2:** Risk of neoplastic disease manifestation in individual organs in cases of hamartomatous polyposis syndromes

**Organ**	**Cumulative risk (%) of cancer development in individual syndromes of hamartomatous polyposis**		
	**Juvenile polyposis syndrome**	**Peutz - Jeghers` syndrome**	***PHTS***
Thyroid gland			3-10
Breasts		(8% risk by age 40, 45% by age 70).	25-50
Stomach	21	29	
Pancreas	Two cases	36	
Small intestine		13	
Large bowel	9%-68% (17%-22% by age 25	57 (9% ba age 40)	
Kidneys			2
Urinary bladder			3
Uterus		9	6
Uterine cervix		10	3
Ovaries		21	2
Testes		9	

Individual syndromes of hamartomatous polyposis frequently manifest similar /symptoms, particularly during the early stage of the diseases when in several cases their clinical pictures do not allow for a differential diagnosis [2]. The correct diagnosis of the disease using molecular methods allows treatment to be implemented earlier and therefore more effectively since it is followed by a strict monitoring of organs that manifest a predisposition for neoplastic transformation [3].

### Juvenile polyposis

Juvenile polyposis (MIM # 174900) is a rare disease inherited in an autosomal dominant manner, described by McColl in 1966 [4]. It occurs at an incidence of 1 per 100,000 births [5]. In most of the recorded cases juvenile polyposis manifests a familiar involvement. Its diagnosis is based on the detection of polyps, histopathologically defined as juvenile polyps. Juvenile polyps are characterized by hyperplasia of mucous glands, retention cysts accompanied by oedema, emboli in gland openings, rich lamina propria of the mucosa with an absence of smooth muscles, inflammatory infiltrates and a prevalence of stroma (Table 3) [6]. The diameter of the polyps ranges between 1 millimeter and a few centimeters. Polyps most commonly occur in the large bowel and the anus (80%), although they can appear in the upper part of the digestive tract, in the stomach and in the small bowel. Single polyps are manifested in 75% of the patients but the occurrence of multiple juvenile polyps can also be noted. In respect to the number of polyps, variability can be noted even in members of a single family. Single juvenile polyps are detected in around 2% of children and adolescents but they display no malignant potential [7]. On the other hand, among patients with juvenile polyposis the risk of malignancy is much higher: according to various papers on the subject it ranges from nine to over fifty percent of the cases.

**Table 3:** **Diagnostic criteria for recognising hamartomatic polyposities**[8,47]

**Syndrome of hamartomatous polyposities**	**Diagnostic criteria**
Juvenile polyposis	• Numerous juvenile polyps (at least 3) in the large bowel and the rectum
	• Any number of juvenile polyps in patients with familial course of the disease
	• Juvenile polyps outside the colon (in the stomach or the small bowel) [8].
Peutz–Jeghers syndrome	• Three or more histologically confirmed polyps
	• Any number of polyps characteristic of PJS in patients with a burdened family anamnesis
	• Typical melanotic dermomucosal lesions in patients with a burdened family anamnesis
	• Any number of polyps typical for PJS and typical melanotic dermomucosal lesions
Cowden’s syndrome	**Symptomatic criteria:**
	Dermomucosal lesions
	• Trichilemmal cysts
	• Acral papilla
	• Papillary lesions
	• Lesions in mucous membranes
	**Major criteria:**
	Breast cancer
	Thyroid carcinoma (particularly follicular)
	Macrocephaly (frontal-occipital circumference of the skull ≥ 97 percentiles)
	Cerebellar dysplastic ganglioma
	Endometrial carcinoma
	**Minor criteria:**
	Other thyroid lesions (e.g. enlargement of thyroid gland)
	Mental retardation (IQ ≤ 75), hamartomatous polyps
	Fibrocystic mammary dysplasia
	Adenomas
	Fibromas
	Cancers of urogenital organs
Syndrome of mixed polyposity	Lack of defined diagnostic criteria for the syndrome
	Diagnosis is based on manifestation of numerous polyps of a variable histopathological type

Juvenile polyposis is diagnosed according the following criteria [8]:

– at least 3 polyps detected on colonoscopy

– juvenile polyps in the entire digestive tract

– in cases of family history of the disease any number of juvenile polyps.

Three categories of the disease are distinguished [9,10]:

– juvenile polyposis of infants

– juvenile polyposis of the large bowel

– general form of juvenile polyposis.

The difference between juvenile polyposis of the large bowel and the general form of juvenile polyposis depends on the localization of the polyps. It is estimated that in over 20% patients with JPS inborn errors are detected in various organs. In the alimentary tract Meckel’s diverticula with umbilical fistula andmalrotation of small intestine have been detected. Cases of undescended testes, unilateral renal agenesia and split uterus were diagnosed in the urogenital system. Inborn errors in the chest include a defect in the interatrial septum, arterionevous haemangiomas, stenosis of the pulmonary valve, Fallot’s tetralogy, aortal stenosis, and persisting arterial duct. Macrocephaly, a communicating hydrocephalus and rachischisis were found within the central nervous system. Moreover, osteomas, mesenteric haemangiomas, inherited teleangiectasias, hypertelorism, inborn amniotony, supernumerary toes and acute intermittent porphyria have also been seen.

The manifestation of juvenile polyposis is preconditioned by mutations in *SMAD4* and *BMPR1A* genes [5,11,12].

Gene *BMPR1Ac* (OMIM *601299; *Bone Morphogenetic Protein Receptor, Type IA*) resides in chromosome 10, in q22-23. The gene consists of 11 exons. It codes for a protein of 532 amino acids, belonging to the family of TGF-β/BMP, representing a type I receptor with properties of serine-threonine kinase [13]. The transcript of *BMPR1A* gene includes 3,613 nucleotides [14,15]. It undergoes expression in almost all tissues including the skeletal muscles, less intensely in the heart and placenta.

The *SMAD4* (OMIM*600993, *mothers against decapentaplegic, drosophila, homolog of, 4*) gene is located in chromosome 18, in the region of q21.1. It consists of 11 exons. The genomic sequence of the gene includes 50 thousand base pairs and its respective mRNA consists of 3,197 nucleotides. It codes for a protein including 552 amino acids. *SMAD4* is a suppressor gene and it participates in the passage of a signal along the pathway of transforming growth factor β (TGF β) and its ligands [13]. *SMAD4* is included among the “common” SMAD, it contains two conserved domains of MH1 and MH2 (*Mad Homology domain*). The amine terminus of the SMAD terminus ends with an MH1 hair-pin domain, demonstrating DNA-binding activity. The carboxy terminus of SMAD proteins ends with a strongly conserved domain of MH2. It is responsible for interaction with proteins involved in the translocation of the complex to cell nucleus and interaction with DNA-binding cofactors [16]. The linker of Co-SMAD contains a leucine-rich NES (*nuclear export signal*) recognized by CMR1. Interaction of *SMAD4* with phosphorylated Co-SMAD masks NES, protecting *SMAD4* from recognition by CMR1 and from export to the cell nucleus. Dephosphorylation of receptor SMAD and dissociation of the complex allows *SMAD4* to be exported . The import of SMAD proteins to the cell nucleus develops with no involvement of nuclear transport factors. Such an importin-independent transport has also been described in cases of components participating in other transformations, e.g. in the case of β-kateniny along the pathway of *Wnt*. This is possible due to the direct interaction of SMAD with nucleoporins, mediated by interaction of the hydrophobic corridor in the MH2 domain with the region of FG repetitions in nucleoporins [17].

The phosphorylated SMAD bind with Co-SMAD, i.e. *SMAD4*. The complex formed in this way passes to the cell nucleus where it becomes involved in the control of expression including several genes, as either positive or negative regulators of the alterations [18,19]. Activation as well as repression requires the participation of the same SMAD proteins while cell-specific interaction with factors serving as coactivators or corepressors shapes the appropriate response. The complex of *SMAD4* with R-SMAD binds to DNA through the MH1 domain, recognizing the palindrome DNA sequence of GTCTAGAC. Such an SMAD-binding sequence (SBE) is frequently noted in genes which undergo expression in the presence of TGF β/BMP ligands. GTCTAGAC SBE is present, on average, in every 1,024 base pairs in the genome or at least one such sequence can be noted in a control region of every moderate size gene [17]. The literature of the subject describes three mechanisms in which transcription can be modified by SMAD and other transcription factors acting on a promotor or enhancer [16]. The first mechanism involves binding of the active R-SMAD and Co-SMAD complex to a transcription factor and such a multi-molecular complex binds to a recognized sequence of DNA. Another mechanism involves the separate binding of SMAD and a cofactor to DNA; the interaction of the two proteins stabilizes the enhancer properties. The last manner of control includes the independent () binding of SMAD and the additional factor to a specific site of DNA. They act separately but in a synergistic manner.

Mutations in the *SMAD4* gene have been noted in around 20% of patients with familial juvenile polyposis [11,20] while a similar incidence of mutations has been detected in the *BMPR1A* gene. In these genes over 120 mutations have been detected as leading to the development of polyps linked to juvenile polyposis syndrome. The mutations include first of all, small alterations, point mutations and small deletions. Nevertheless, a high proportion of alterations detected in patients with juvenile polyposis also involve extensive lesions. Large deletions were observed in the region of q22-q23 of chromosome 10. The alterations affected the two neighbouring genes of *PTEN* and *BMPR1A*. Mutations in these genes are engaged in the development of distinct syndromes of hamartomatous polyposis.

The mutations that have been described so far are of a heterogenous character, with the exception of a single mutation c.1244_1247delAGAC in exon 9 of the *SMAD4* gene. This mutation is located in a hot spot, a region containing four binucleotide repetitions of AG, where the looping off of a DNA strand fragment probably takes place, which undergoes a deletion.

Certain correlations were detected between phonotype and genotype in JPS patients carrying a mutation in the *SMAD4* gene, who were found with higher frequency to carry large polyps in their stomachs. Germ-line mutations in the *SMAD4* gene are responsible for a more aggressive phenotype of juvenile intestinal polyposis, manifesting itself in the form of a vascular malformation within sublayer components when the mutation was located before the codon of 423. It was also noted that polyps with mutation in the *SMAD4* gene are detected in both upper and lower parts of the digestive tract while polyps with mutations in *BMPR1A* gene are restricted to the rectum and the anal canal.

### Peutz-Jeghers` syndrome

Peutz - Jeghers syndrome (PJS; OMIM 175200) is inherited in an autosomal dominant way. The first description of the syndrome was published by L.A.Peutz in 1921, 28 years later H. Jeghers described the clinical presentation of the disease in detail. The first signs in the form of hamartomatous polyps and pigment skin lesions appear in childhood. Incidence of the syndrome ranges from 1/29,000 to 1/120,000 births. The polyps appear during the second or third decade of life in 80-100% of patients. They may be located all along the alimentary tract although the frequency of their manifestation is variable and depends on localization within the digestive tract (Table 3). They are most frequently detected in the small bowel (96%), followed by those in the colon and the stomach. In histopathology they are present as tree-resembling branches of smooth muscle bundles. The core of the polyps is formed by connective tissue and smooth muscles. The entire lesion is covered by normal looking epithelium. In the patients benign polyps were detected outside the digestive tract, in the nose, the respiratory tract, the gallbladder and the urinary bladder. They are multiple and their size ranges from 1 to 3 cm. The risk of developing intestinal cancer in patients with Peutz – Jeghers` syndrome is slighly higher than that in the general population. However, it should be noted that hamartomatous polyps, particularly multiple ones, may result in several complaints from the digestive tract. They may cause ileus (due to intussusception) and bleeding from the lower part of the tract, due to the ease of polyp autoamputation [21,22]. Papers on patients with Peutz - Jeghers syndrome describe some cases of extraintestinal malignancies [23,24]. An elevated risk was noted for development of cancers in the pancreas, the breasts, the ovary and the uterus [25]. Another characteristic sign of the disease is the development of muco-cutaneous melanosis, which appears in infancy or in early childhood. The dark-brown, black or blue spots of 1–5 mm in size are manifested in over 90% of patients. They may develop around the mouth, the nostrils, the eyes, the cheeks, on the tongue or the palate. Cases were also described in which melanosis appeared on the hands, feet, around the umbilicus or in the perianal region. Following pubescence and during adulthood the spots may turn pale. Diagnosis of Peutz Jeghers` polyposis is based on clinical signs, in cases of patients with a familial manifestation of the disease the criteria for diagnosis are restricted to the detection of melanin deposits. In the absence of familial anamnesis it is necessary to confirm the manifestation of at least two hamartomatous polyps.

Peutz - Jeghers syndrome is preconditioned by the manifestation of mutations in the *STK11* (OMIM*602216 Serine/Threonine Protein Kinase 11) gene, located in the short arm of chromosome 19, in the 13.3 region. The gene consists of 10 exons, of which 9 code for a protein. It undergoes comprehensive expression during embryonal development but also in the organs of adults, particularly in the pancreas, the liver and skeletal muscles. The *STK11* protein consists of three main domains, the N-terminal non-catalytic domain with two signals for nuclear localization, the highly conserved kinase domain and the regulatory domain at the carboxy end. The kinase domain of this 433 amino acid protein is located between the 49th and 309th amino acid. The *STK11* protein contains a few sites which undergo phosphorylation and prenylation and the nuclear localization signal (NLS). Due to the activity of kinases serins are phosphorylated at positions of 31 and 325 and threonine at position 363. *STK11* is also capable of undergoing phosphorylation on threonins at positions 185, 189, 336, and on serine at position 402. Autophosphorylation of *STK11* at position Thr189 is very important for kinase activity of the protein. On the other hand, the prenylation motive of Cys^430^-Lys-Gln-Gln^433^ is positioned at the carboxy terminus of the protein. Loss of *STK11* protein function precipitates the development of various defects. This reflects the fact that the *STK11* protein is involved in a number of important cellular processes. In *Xenopus*, a homologue of the *STK11* gene, XEEX1, is engaged in the process of early embryonal development. On the other hand, mice lacking the *STK11* gene die at around the 8th day of embryogenesis. In the case of *STK11+/−* mice, manifestation of polyps is observed, which in histopathology are very similar to those noted in PJS. Molecular analysis showed that loss of a single *STK11* allele is sufficient for the development of the polyps. Following the 45th week of life in >70% male *STK11+/−* mice and in 20% female *STK11+/−* mice histopathologically variable types of liver malignancies are seen. The cancer cells showeda loss of both *STK11*[26,27]. *STK11* was found to control the TGF β pathway, forming a complex with the *SMAD4* protein through LIP1. LIP1 forms a specific bridge between the two proteins [28]. *STK11* also interacts with the *PTEN* protein [29]. Moreover, *STK11* kinase participates in p53-dependent apoptosis.

Germ-line mutations of *STK11* have been detected in 70% patients with an inherited form of the disease. In cases of patients with negative anamnesis` detectability of the mutations approximates 50% [30]. In the *STK11* gene over 230 mutations have been described so far, including 70 point mutations. A significant portion of the mutations include small deletions (54) and small insertions (33). Nevertheless, large deletions, including individual exons or even deletions of the entire gene are also frequent in patients with PJS [31].

### Cowden’s syndrome

Cowden’s syndrome (OMIM #158350; or *Cowden Disease*; CD) is a very rare syndrome of hamartomatous polyposis. Its incidence is estimated at 1 per 200,000 deliveries. Its typical trait involves various hamartomatous lesions in tissues originating from three germ layers: the endoderm, ectoderm and mesoderm. Apart from the gastrointestinal region (71% of the patients), the lesions are manifested in the skin, mucous membranes and other organs. An international consortium dealing with Cowden’s syndrome evaluated a diagnostic criteria, which includes dermomucosal lesions, including fibromas of the oral cavity and papillary and hyperkeratotic alterations on the face and extremities [32]. In almost 99% of patients dermomucosal lesions develop before the 30th year of age. Within the alimentary tract hamartomatous lesions develop all along the tract, being most frequent in the stomach, the colon and the oesophagus [33]. In the oesophagus they appear as glycogenic keratinization. In this syndrome hamartomatous lesions include, first of all, polyps, adipomas and gangliomas [34,35]. In Cowden’s syndrome polyps are distinguished by the presence of nerve elements, not seen in the other syndromes of hamartomatous polyposis. Moreover, in patients with Cowden’s syndrome defects of the central nervous system, such as macrocephaly (38%), mental retardation, Lhermitte-Dulclos disease (LDD) and cerebellar gangiomas can be detected. Defects of eyes and arterio-venous developmental lesions are also noted [36-38]. Defects of bones in the skull, the spinal column and the hands trouble every third patient. Patients with the syndrome are burdened with an increased risk of developing benign or malignant tumours in the thyroid gland, the lungs, the kidneys, the retina, the breasts, the uterus and the skin. Breast cancers are detected in 30% to 50% of women with Cowden’s syndrome and in 25% the cancer is bilateral. In such patients breast cancer develops at the very young age, approximately 10 years earlier than in the general population [36]. The risk of developing thyroid cancer in women and men with Cowden’s syndrome is 10% higher than in the general population. In respect to histopathology the most frequent thyroid cancer in patients with Cowden’s syndrome is papillary or follicular but individual cases were also noted of medullary thyroid carcinoma [39]. The *PTEN* (OMIM*601728; *Phosphatase And Tensin Homolog*; *PTEN*) gene, responsible for the development of Cowden’s syndrome was mapped in 1997 on chromosome 10, region q23. This is a suppressor gene, coding for a protein consisting of 403 amino acids, representing a phosphatase responsible for the removal of phosphate groups from molecules. Definition of *PTEN* crystalline structure showed that the N-terminal domain of the phosphatase strictly adheres to the C2 domain at its carboxy terminus. The two domains create a basic catalytic unit, encompassing the almost entire peptide sequence of the protein, with the exception of the small tail in the N-terminal portion of the protein and a longer 50 amino acid-fragment at the carboxy terminus. The domain of phosphatase and C2 creating the catalytic core of the protein are sufficient for its normal function. The remaining parts seem to be involved in the control of activity and/or interaction of *PTEN* with the other molecules [40]. *PTEN* can de-phosphorylate both proteins and lipids of cell membrane. It removes the phosphate group from inositol ring position D3, from 3,4,5 phosphatidylinositol triphosphate and 3,4 phosphatidylinositol diphosphate, produced during the transmission of cellular signals through the activity of phosphoinositol 3' kinase (*PI3K*) [41]. *PTEN* acts as a specific switch-off for signal transmission along the PI3K pathway, and in this way stops the cell cycle at the G1 phase. The activity of the *PTEN* gene, involving antagonization of phosphoinositol 3’ kinase action, inhibits the activity of multiple oncoproteins exerting their effect through the PI3K kinase. *PTEN*-PI3K controls fundamental cellular processes linked to the mechanism of neoplastic transformation. *PTEN* participates in the control of the cell cycle through Akt kinase. Among substrates of Akt kinase which play a significant role in the cell cycle transcriptional factors such as *FKHR* (Forkhead transcription factor), *AFX*, *FKHRL1* or *GSK3* can be distinguished. *PTEN* also controls cell divisions. In pten−/− fibroblasts the response to stimulation of apoptosis is lowered due to the augmented transcription of proapoptotic genes, i.e. FAS and Bim. The activity of *PTEN* protein phosphatase leads to the inhibition of FAK (*Focal Adhesion Kinase*), responsible for cellular adhesion and the capacity of cells to migrate [42]. *PTEN* protein plays also an important role in the process of angiogenesis and participates in control over the mTOR pathway. In *D. melanogaster* loss of the *dpten* gene function results in an increased size of cells and organs while overexpression of *dpten* in yeasts causes an inverse phenotypic effect. In the case of mice loss of the *pten* gene in neurons leads to the development of a set of traits resembling those present in Lhermitte-Duclos disease, representing one of the clinical presentations of Cowden’s syndrome [43].

Somatic mutations in the *PTEN* gene lead to the development of a number of various neoplasms. They are detected in 80% of patients with Cowden’s syndrome. Mutations in the *PTEN* gene are also detected in other syndromes of hamartomatous polyposity, among others in Bannayan-Riley-Ruvalcaba’s syndrome. The frequency of lesions being detected in the *PTEN* gene approaches 60%. In the computer base of mutations 208 mutations are described in the *PTEN* gene, of which the majority involved mutations of altered sense and nonsense mutations (87 mutations), small deletions and insertions (75 mutations) [44]. In patients with Cowden’s syndrome mutations in the *PTEN* gene are noted in the promoter region while deletions of a portion or entire gene are commonly observed in patients with Bannayan-Riley-Ruvalcaba’s syndrome.

### Hereditary mixed polyposis syndrome

The morbid unit (MIM #610069, Hereditary Mixed Polyposis Syndrome, HMPS) first appeared in literature in 1971: the case was described of an 11-year-old girl with juvenile polyps and adenomas in the colon and small intestine. However, it was not until 1987 that Sarles suggested the term of mixed polyposis describing the cases of a father and son with numerous various types of polyps in the colon. In the father they included metaplastic polyps and adenomas, in the son juvenile polyps were additionally diagnosed. In the most accurate way clinical traits of mixed polyposity syndrome were presented in a family of a few generations, termed SM96 [45,46]. Among more than 200 members of the family 42 individuals demonstrated the presence of various types of polyps, ranging from tubular adenomas, papillary adenomas, flat adenomas to hyperplastic polyps and atypical juvenile polyps. Histologically, the atypical juvenile polyps carried traits of hyperplastic polyps and of adenomas. Colonoscopic tests were used to demonstrate more than ten polyps in the colon and the anus. The average age of the patients diagnosed with HMPS in the SM96 family was 40 years.

Cao presented two three-generational families with a course of the disease very similar to that of the SM96 family. In most of the cases polyps were located in the large bowel. In those family members Cao noted no extraintestinal lesions.

Individuals with HMPS were found to manifest an augmented predisposition to the development of malignancies in the large bowel.

The gene responsible for the development of mixed polyposity has not yet been identified. Analysis of linkages defined the region strictly bound to the appearance of the morbid signs. This was termed CRAC1 and it is located on chromosome 15, on its long arm. In a proband in one of the families presented by Cao a heterozygous mutation was disclosed, a deletion of 11 nucleotides in exon 2 of the *BMPR1A* gene. In the remaining family members the mutation could not be detected. Mixed polyposis is the least recognised disease among the hamartomatous polyposity syndromes and it still requires further experimental studies, which would lead to a rapid and simple diagnosis.

### Care of patients with hamartomatous syndromes

Morbid syndromes linked to hamartomatous polyposities form a rather heterogenous group both in respect to the number and localization of polyps and the risk of cancer development in the alimentary tract and other organs. Even if the diseases are not among the most frequently occurring ones they remain dangerous to patients not only due to their predisposition to manifest themselves as a neoplastic disease but also due to non-neoplastic signs, such as haemorrhages, intussusception and ileus. Every hamartomatous polyposis syndrome manifests its own organ-specific localization of /symptom manifestation and, consequently , each of them requires a different strategy of diagnostic management. Therefore, an accurate qualification of the patients to a specific morbid syndrome represents a basic step towards the appropriate care of patients affected by the predispositions. Recommendations related to diagnostic studies in hamartomatic polyposities are based only on the opinions of experts. To date, no randomised diagnostic studies have been conducted on the efficacy of medical care programmes in the diseases [47]. For Peutz - Jeghers syndrome diagnostic care is directed to the organs endangered with neoplastic transformation which vary depending on the gender of the patient. In both males and females examination of the small bowel is recommended (small intestine passage) beginning at the 8th year of age, every two-three years. Reports are available on the advantageous role of capsule endoscopy but the number of tests that have been performed is still insufficient to recommend the approach in the form of management recommendations [48]. Extensive diagnostic and therapeutic hopes may be founded on double-balloon endoscopy, allowing unitemporal polypectomies in necessary cases [49]. Colonoscopy is recommended every 2–3 years up to the 18th year of life. It should be stressed that endoscopic surveillance with regular polypectomies seem to effectively safeguard the patients from the development of malignant tumours [50].

Beginning at the 24th year of age it is also recommend to subject the patient every 1–2 years to a USG examination of the pancreas. In women the tests include monthly self-examination of breasts from 18 years of age onwards and an according to international guidelines for hereditary breast cancer, annual breast ultrasound and MRI from age 25 and annual mammography from age 35 [8,47]. The ovaries should be examined once a year between birth and the 12th year of age and, then, from the 21st year of age. In male patients testes should be tested from birth till the 12th year of age [47,51]. In juvenile polyposity the care of patients focuses on monitoring the alimentary system. However, in a few families co-manifestation of a haemorrhagic angiomatosis has been observed in cases of mutations in the *SMAD4* so the potential for aberrant vascular development should be taken into account. Attention should be paid to the occurrence of heamorrhages, anaemia, abdominal pains, diarrhea and alterations in the shape or colour of foeces in the patients. Manifestation of such alterations indicates the necessity of performing additional studies, including colonoscopy. In asymptomatic patients endoscopic tests (gastroduodenoscopy and colonoscopy) should be conducted beginning from the 15th year of age. If endoscopy discloses the presence of polyps they should be removed and, in such a situation the test should be repeated and polyps should be removed every year [47]. If no polyps are detected the test may be repeated once every three years. In mixed polyposity endoscopic examination of the large bowel is recommended once a year and the diagnosed polyps should be removed by polypectomy [51,52]. Cowden’s syndrome carries the risk of a neoplastic disease in various organs, therefore, prophylactic examinations should include the thyroid gland, the breasts and endometrium. No specific recommendations are available for testing the alimentary tract but certain authors recommend a periodical examination of the tract with radiologic studies [3,47,53]. Examination of the breasts bay self-examination conducted once a month and annual breast ultrasound and MRI should begin at the 30th year. The annual mammography should be performed from age 35. Thyroid USG examination should be supplemented with an aspiration thin-needle biopsy of detected tumours [3,53].

The basic method in the care of patients with the above polyposis syndromes involves an endoscopic surveillance with the regular removal of qualifying polyps (large, with macroscopic signs of malignancy, contact or spontaneous bleeding). In cases of a severe course, with a high number of rapidly growing polyps in the colon, a colectomy with ileo-rectal anastomosis may be recommended [3]. If a great number of polyps, difficult to remove, develop in the rectum, restorative proctocolectomy should be considered.

## Competing interests

The authors declare that they have no competing interests.

## Authors' contributions

All authors contributed to the literature search and manuscript preparation. All authors read and approved the final manuscript.
